# Phase i study evaluating the treatment of patients with hepatocellular carcinoma (HCC) with carbon ion radiotherapy: The PROMETHEUS-01 trial

**DOI:** 10.1186/1471-2407-11-67

**Published:** 2011-02-12

**Authors:** Stephanie E Combs, Daniel Habermehl, Tom Ganten, Jan Schmidt, Lutz Edler, Iris Burkholder, Oliver Jäkel, Thomas Haberer, Jürgen Debus

**Affiliations:** 1 Department of Radiation Oncology, University Hospital of Heidelberg, Im Neuenheimer Feld 400, 69120 Heidelberg, Germany; 2 Department of Gastroenterology, University Hospital of Heidelberg, Im Neuenheimer Feld 410, 69120 Heidelberg, Germany; 3 Department of Surgery, University Hospital of Heidelberg, Im Neuenheimer Feld 110, 69120 Heidelberg, Germany; 4 German Cancer Research Center (dkfz), Department of Biostatistics, Im Neuenheimer Feld 290, 69120 Heidelberg, Germany; 5 Statistische und Biometrische Lösungen, Pistorstr. 7, 66482 Zweibrücken, Germany; 6 Heidelberger Ionenstrahl Therapiezentrum (HIT), Im Neuenheimer Feld 450, 69120 Heidelberg, Germany

## Abstract

**Background:**

Treatment options for patients with advanced hepatocellular carcinoma (HCC) are often limited. In most cases, they are not amenable to local therapies including surgery or radiofrequency ablation. The multi-kinase inhibitor sorafenib has shown to increase overall survival in this patient group for about 3 months.

Radiation therapy is a treatment alternative, however, high local doses are required for long-term local control. However, due to the relatively low radiation tolerance of liver normal tissue, even using stereotactic techniques, delivery of sufficient doses for successful local tumor control has not be achieved to date.

Carbon ions offer physical and biological characteristics. Due to their inverted dose profile and the high local dose deposition within the Bragg peak precise dose application and sparing of normal tissue is possible. Moreover, in comparison to photons, carbon ions offer an increased relative biological effectiveness (RBE), which can be calculated between 2 and 3 depending on the HCC cell line as well as the endpoint analyzed.

Japanese Data on the evaluation of carbon ion radiation therapy showed promising results for patients with HCC.

**Methods/Design:**

In the current Phase I-PROMETHEUS-01-Study, carbon ion radiotherapy will be evaluated for patients with advanced HCC. The study will be performed as a dose-escalation study evaluating the optimal carbon ion dose with respect to toxicity and tumor control.

Primary endpoint is toxicity, secondary endpoint is progression-free survival and response.

**Discussion:**

The Prometheus-01 trial ist the first trial evaluating carbon ion radiotherapy delivered by intensity-modulated rasterscanning for the treatment of HCC. Within this Phase I dose escalation study, the optimal dose of carbon ion radiotherapy will be determined.

**Trial registration:**

NCT 01167374

## Background

Primary liver cancer is the fifth most common neoplasm in the world, and the third most common cause of cancer-related death; of those, hepatocellular carcinoma (HCC) amounts to about 75-90% [1]. More than 600 000 new cases are diagnosed yearly, with and incidence of 5.5 to 14.9 per 100 000 [2,3]. In some areas of Asia, HCC is endemic, and is the most common cause of death due to cancer [4,5]. In Europe and the USA, HCC has shown an increase in incidence over the last years, which is attributed to an increase in Hepatitis C-Virus (HCV) infections [6-10]; other main risk factors include cirrhosis, alcohol or haemochromatosis [11].

It is known, that in Europe, North America and Japan, HCV-related HCC represents about 75% of all cases, where as in the Asia-Pacific Region (excluding Japan) 70% of all patients with HCC are diagnosed with chronic Hepatitis-B-Virus (HBV)-infections [12].

In Western countries, about 30-40% of all patients with HCC are diagnosed with early stages of the disease and potentially curative treatments such as surgical resection, liver transplantation (TPX) or locoregional procedures such as radiofrequency ablation of chemoembolisation can be performed, depending on liver function and tumor burden. In well selected patient groups, overall survival (OS) rates of 60 - 70% at 5 years have been observed [13].

However, treatment options for patients with advanced staged HCC are limited and this patient group is associated with a poor prognosis [13]. In the past, no systemic chemotherapeutic treatment regimen has improved outcome in patients with advanced HCC [14-16]. New substances addressing single molecular targets, such as EGFR-inhibition with cetuximab, have not demonstrated convincing results [17]. Only recently, however, the multi-kinase inhibitor sorafenib (Nexavar^®^) has been shown to be effective in patients with HCC: In the multicenter SHARP trial (Sorafenib Hepatocellular Carcinoma Assessment Randomized Protocol), an increase in OS in patients with advanced HCC from a median of 7.9 months in the placebo group to 10.7 months in the sorafenib group could be demonstrated [18]. The study included 602 patients in a double-blind setting, and treatment with sorafenib was performed with 400 mg bid or placebo. Although OS and time to radiological progression were increased significantly, time to symptomatic progression (determined by the Functional Assessment of Cancer Therapy-Hepatobiliary Syndrom Index 8 (FHSI8) questionnaire or the occurrence of unacceptable adverse events or death) was not changed. Moreover, according to the RECIST-criteria, most controlled patients only showed stable disease in imaging, and only 2 out of 229 patients treated with sorafenib showed a partial response of the HCC; no complete remissions were observed.

Another randomized phase III trial evaluating efficacy and safety of sorafenib in patients with advanced HCC was performed in the Asia-Pacific region, and also demonstrated good tolerability of sorafenib and an increase in OS from 4.2 months to 6.5 months [19]. Again, time to symptomatic progression was unaltered by sorafenib, and the vast majority of patients controlled with sorafenib showed stable disease, with only 5 out of 150 partial responses and no complete remissions.

Therefore, this group of patients with advanced HCC is still characterized by unsatisfactory outcome, and treatment optimization is needed.

External Beam Radiotherapy has been applied for the treatment of HCC in the past with only moderately convincing clinical results. With conventional photon techniques, dose tolerance of surrounding normal liver tissue has been a major problem and has limited application of high local doses to larger liver tumors. Radiation induced liver disease (RILD), which is defined as a veno-occlusive disease leading to development of ascites, icterus, increase in liver enzymes and hepato-encephalopathy, is known to develop with total doses of 30-35 Gy delivered to the total liver [20]. However, more conformal radiation techniques have been shown to be safe, however only moderately effective, in patients with HCC, mainly due to the known dose-response-relationship of liver tumors [20-22]. Park et al. could show that patients treated with total doses of > 50 Gy showed significantly higher outcome and increased rates of partial tumor responses after radiotherapy in 158 patients with HCC treated with 3D-conformal radiotherapy with increasing doses [23]. Among other studies, this group could show that dose was the only reproducible prognostic factor for tumor control [23,24]. However, as total doses are increased even using 3D-conformal photon techniques, the rates of radiation-induced side effects increase, and therefore application of higher and locally effective radiation doses are limited especially in larger tumors.

Using stereotactic photon treatments, functional normal liver tissue may be spared more effectively. In the past, several groups have implemented extracranial stereotactic treatment for liver tumors in single-dose or hypofractionated settings, however, with increasing tumor sizes which is common in HCC, dose application is again limited [25-28].

Therefore, treatment of patients with advanced stage HCC is often limited to systemic treatment with sorafenib.

However, new radiation modalities such as particle therapy offer distinct physical and biological characteristics and are a promising treatment alternative for patients with HCC.

Heavy charged particles provide the physical advantage of an inverted dose profile which enables steep dose gradients. Neighboring organs at risk and surrounding normal tissue can be spared from radiation doses. Additionally, carbon ions, as high-LET beams, are characterized by an enhanced relative biological effectiveness (RBE). For hepatocellular carcinoma cell lines, RBE values between 2 and 3 have been reported depending on cell line and endpoint [29,30]. In general, high local doses are required for long-term local tumor control in patients with HCC. The distinct physical dose characteristics of particle therapy allow the application of higher local doses while sparing healthy normal tissue, which was a limiting factor in the application of radiation doses for the treatments of HCC with conventional radiation techniques. Results of proton radiotherapy in patients with HCC reported from Japan are promising, even in patients with advanced tumors [31-37]. Carbon ions offer, additionally to the same physical benefits, an increase relative biological effectiveness, which has been shown to be beneficial with respect to outcome in several different tumor entities [38]. Results from carbon ion radiotherapy for the treatment of patients with HCC have shown safety and high local control and response rates [38-40].

In Chiba and Tsukuba, researchers have evaluated ion beam radiotherapy in patients with HCC showing promising results. Kawashima and colleagues treated 30 patients with solitary HCC not amenable to surgery or local ablation therapy with protons with total doses of 76 Gy E in single fractions of 3.8 Gy E [33]. All patients had liver cirrhoses, which was Child A in 20 and Child B in 10 patients. No severe side effects were observed, and 2-year actuarial local progression-free rate was 96%, with an actuarial OS rate of 66%. Out of 30 patients, 24 developed a complete response of the HCC. The Tsukuba group demonstrated local control of 93% after 5 years in patients with HCC with limited treatment options other than radiotherapy [35]. In this study, total doses of 63-84 Gy E proton radiotherapy were applied in a fractionated regimen using 13 to 27 fractions. OS rates were 62% and 33% at 2 and 5 years. No toxicities ≥ Grade III were observed, and treatment was well tolerated. The same group could also demonstrate that high-dose proton radiotherapy may be applied safely in patients with HCC associated with a portal vein tumor thrombus, severe liver cirrhosis or even in the aged patient population [31,34,37].

Carbon ions additionally offer a higher biological effectiveness due to the characteristic and severe radiation damages produces in target tissues. Is has been shown that carbon ion radiotherapy leads to an increased in local control especially in radiation resistant tumors, such as chordomas and chondrosarcomas, adenoidcystic carcinomas, melanomas as well as HCC [38]. Kato an colleagues treated 24 patients with HCC with carbon ion radiotherapy in a dose-escalation study increasing total doses from 49.5 Gy E to 79.5 Gy E in dose increments of 10% in a fixed 15 fraction setting [39,40]. It could be shown that dose increase was safe without severe treatment associated side effects, even in the highest dose groups. The optimal dose was determined to be 72 Gy E, which was the lowest dose to show the highest local tumor control without grade III toxicity. In this group, the overall tumor response rated was 71%, with a complete response in 10 out of 24 patients, and a partial response on 7 out of 24 patients.

Although patient collectives in these studies on particle therapy and on the treatment of patients with sorafenib are heterogeneneous, the data show that particle therapy may be a successful treatment alternative for patients with advanced HCC.

In the present PROMETHEUS-01 trial, carbon ion radiotherapy will be evaluated in patients with advanced HCC.

Since 1997, carbon ion radiation therapy is available by the Department of Radiation Oncology, University Hospital of Heidelberg, at the Gesellschaft für Schwerionenforschung in Darmstadt, Germany. Clinical studies on skull base tumors, brain tumors as well as head-and-neck-tumors with skull base invasion treatment safety could be demonstrated. Based on the clinical results, the Heidelberg Ion Therapy Center (HIT) was built delivering ion beams with equivalent technology. Worldwide, particle therapy is available in the clinical routine in a large number of centers.

At the University of Heidelberg, patients with HCC are treated in the interdisciplinary setting consisting of visceral surgery, internal medicine/gastroenterology, radiology and radiation oncology. Therefore, patients will be provided the best possible oncological care on a professional basis.

## Methods/Design

### Study design

The trial will be performed as a single-center Phase I Dose Finding study.

Patients fulfilling the inclusion criteria will be included into the following Phase I dose escalation treatment scheme shown in Table 1.

**Table 1:** Treatment scheme for dose escalation.

Step 1:	4 × 10 Gy E	40 Gy E
Step 2:	4 × 11 Gy E	44 Gy E

Step 3:	4 × 12Gy E	48 Gy E

Step 4:	4 × 13 Gy E	52 Gy E

Step 5:	4 × 14 Gy E	56 Gy E

### Trial Design

**Figure 1:**
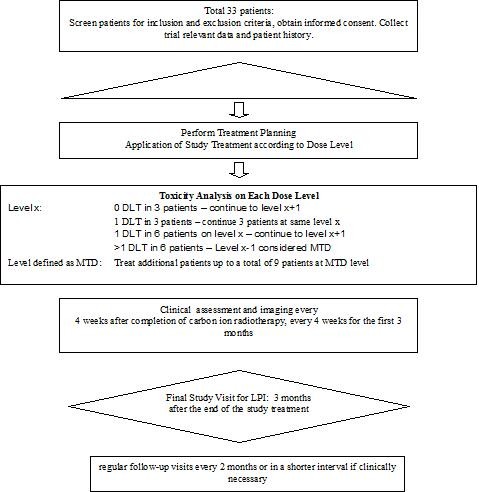
**Flow chart of the Prometheus-01 study**.

The study is performed as a one-armed single center Phase I trial. The treatment schedule is shown in the study flow chart in Figure 1.

### Study objectives

The purpose of the trial is to evaluate carbon ion radiotherapy in patients with advanced HCC. With respect to toxicity, the optimal dose of carbon ion radiotherapy will be determined. Therefore, the primary endpoint is toxicity, secondary endpoints are evaluation of progression-free survival, response, and overall survival after carbon ion radiotherapy.

Focus of the analysis is to evaluate safety and efficacy of carbon ion radiotherapy in patients with HCC. Therefore, the aim of the trial is to observe low rates of toxicity with high local doses due to effect of the altered biology of carbon ions on HCC as well as the superior physical characteristics.

#### Primary Objective

The primary objective is toxicity of carbon ion radiotherapy and the definition of a MTD for subsequent clinical investigation of carbon ion radiotherapy.

#### Secondary Objectives

The secondary objectives of the study are imaging response and progression-free survival.

### Patient selection: Inclusion criteria

Patients meeting all of the following criteria will be considered for admission to the trial:

- histologically confirmed HCC or diagnosis of HCC according to AASLD-guidelines

- macroscopic tumor

- liver-confined disease without extrahepatic disease as diagnosed by CT, MRT, ultrasound and bone scan

- minimal distance of tumor edge to the intestines of 1 cm

- age ≥ 18 years of age

- Karnofsky Performance Score ≥60

- For women with childbearing potential, (and men) adequate contraception.

- Ability of subject to understand character and individual consequences of the clinical trial

- Written informed consent (must be available before enrolment in the trial)

### Patient selection: Exclusion criteria

Patients presenting with any of the following criteria will not be included in the trial:

- refusal of the patients to take part in the study

- previous radiotherapy of the hepatobiliary system

- margin of < 1 cm between tumor edge and intestines

- Patients who have not yet recovered from acute toxicities of prior therapies

- Known carcinoma < 2 years ago (excluding Carcinoma in situ of the cervix, basal cell carcinoma, squamous cell carcinoma of the skin) requiring immediate treatment interfering with study therapy

- Pregnant or lactating women

- Participation in another clinical study or observation period of competing trials, respectively

### Treatment Planning

For particle therapy, patients will be immobilized using an individually manufactured body fixation or positioning device including abdominal pressure plates as described in detail previously [26]. For treatment planning, contrast-enhanced CT (3 mm slice thickness) as well as MR-imaging will be performed for optimal target definition. 4D-CT-imaging is considered standard of care for target definition and for position verification during the course of radiotherapy when treating patients with moving organs. Interstitial fiducial markers are standard of care for treatment planning and patient positioning during treatment and may be implanted prior to radiotherapy within clinical routine.

Organs at risk such as the intestines, stomach, lungs, kidney, spleen and spinal will be contoured. Dose constraints of normal tissue will be respected according to Emami et al. [41].

The Gross Tumor Volume (GTV) will be defined as the area of solid macroscopic tumor contrast enhancement on CT and MR-imaging. The Clinical Target Volume (CTV) will be defined as the GTV plus a margin of 5 mm. The Planning Target Volume (PTV) will include the CTV plus a margin of about 1 cm to account for residual organ motion and setup inaccuracies.

Carbon ion RT planning is performed using the treatment planning software PT-Planning (Siemens, Erlangen, Germany) including biologic plan optimization. Biologically effective dose distributions will be calculated using the α/ß ratio for HCC as well as for the endpoint late toxicity.

No interruptions > 4 days between during study treatment are allowed.

Patient positioning directly prior to particle therapy will be evaluated by comparison of x-rays for static images as well as fluoroscopic imaging to determine residual organ motion in comparison to the DRRs. Set up deviations > 3 mm are corrected prior to radiotherapy.

### Dose Prescription Carbon Ion Radiotherapy

The intensity-controlled rasterscanning system will be used for beam application.

Within this dose escalation study, total doses and fractions will be applied according to the dosing scheme shown in Table 1.

Treatment planning aims in the coverage of the PTV by the 90%-isodose line.

Dose specification is based on biologic equivalent dose because of the high relative biologic effectiveness (RBE) of carbon ions, which differs throughout the target volume due to its dependence on various factors. RBE will be calculated at each voxel throughout the target volumes and biological optimization will be performed.

### Trial Duration, Schedule and Follow-up

The primary endpoint is acute toxicity of carbon ion radiotherapy observed within 30 days of study treatment.

Patients are scheduled for follow-up visits every 4 weeks after completion of carbon ion radiotherapy for the first 3 months, thereafter in 2-months intervals or as needed clinically including contrast-enhanced MRI as well as thorough clinical-neurological and haematological assessment. These follow-up visits are in line with standard care outside of clinical trials.

The last patient included into the study will be followed for at least 3 months after study treatment. This is considered the final study visit. All other patients will be followed regularly as described in detail until death or until 3 months after LPI.

The overall duration of the trial is expected to be approximately 24 months.

### Assessment of Efficacy Parameters

#### - Progression-free Survival and Treatment Response -

Efficacy of the treatment will be recorded according to the RECIST-Criteria (Revised Guidelines, Version 1.1, 2009 [42]):

##### Complete Response (CR)

Disappearance of all target lesions.

##### Partial Response (PR)

at least 30% decrease in the sum of diameters of target lesions, taking a reference the baseline sum diameters.

##### Progressive Disease (PD)

At least a 20% increase in the sum of diameters of target lesions, taking as reference the smallest sum on study (this includes the baseline sum if that is the smallest on study). In addition to the relative increase of 20%, the sum must also demonstrate an absolute increase of a t least 5 mm (Note: The appearance of one or more new lesions is also considered progression).

##### Stable Disease (SD)

Neither sufficient shrinkage to qualify for PR nor sufficient increase to qualify for PD, taking as reference the smallest sum diameters while on study.

The principal investigator or study co-ordinator may be contacted for further discussion on a case by case basis.

### Assessment of Safety Parameters

This study will use the International Common Terminology Criteria for Adverse Events (CTCAE) version 4.0 for toxicity and adverse event reporting. A copy or the CTCAE can be accessed from the CTEP home page: http://ctep.cancer.gov/protocolDevelopment/electronic_applications/ctc.htm

Safety and toxicity of the study treatment will be evaluated by clinical examination, haematological evaluation as well as imaging studies (MRI or CT).

### Statistical calculations for trial sample size

#### Study hypothesis

The study is designed to demonstrate that carbon ion radiotherapy is safe for the treatment of HCC. The aim of the study is to evaluate the optimal dose tolerated with respect to toxicity.

Unacceptable toxicity (DLT) is defined as any irreversible grade 4 toxicity during a time frame of 30 days as classified by CTCAE version 4.0.

### Statistical Methods

#### Sample Size Calculation

The calculation of sample size for the PROMETHEUS-01 trial is based on the traditional dose escalation scheme. DLT are considered any irreversible Grade 4 toxicity classified by CTCAE version 4.0 after study treatment.

Patients are treated in cohorts of three each receiving the same dose.

For observation of DLT, patients are observed for 30 days after study treatment. If the last patient of the cohort is observed 30 days after study treatment, patients of the next cohort may be recruited.

If none of the three patients shows a DLT, the next cohort of three patients receives the next higher dose as described above.

Otherwise, if at least one patient of the cohort develops a DLT (1/3), a second cohort of three patients is treated at the same dose level without escalating the dose.

If exactly one out of the six patients (1/6) treated exhibits DLT, the trial continues as planned at the next higher dose level.

If two or more patients out of the six exhibit a DLT, the dose escalation stops at that level and the next lower level is considered as the MTD. When the escalation has stopped, additional patients will be treated at the MTD to a total of nine patients.

This trial is conducted to choose the MTD of carbon ion radiotherapy between five dose levels. Therefore the sample size at maximum is 33 patients (4 dose levels with at maximum 6 patients and 9 patients at the MTD).

#### Analysis Variables

Primary endpoint is the MTD chosen between the five dose limits based on the traditional dose escalation scheme. Any unpredictable of irreversible grade 4 toxicity possibly, probably or definitely related the study treatment occurring 30 days after completion of study treatment is considered as dose limiting toxicity.

Secondary endpoints are the collection of safety data on the dose levels used in this trial as well as response, progression-free survival, and overall survival.

#### Analysis Populations

The Analysis set for primary endpoint consists of all patients treated at least once with the study treatment. A total of five analysis sets (one per dose level) will be considered.

Analysis set for secondary endpoints: All patients will be included for analysis for secondary endpoints treated at least one with the study treatment. Additionally, a total of five analysis sets (one per dose level) will be considered to determine any differences in secondary endpoints between the 5 dose levels.

### Statistical Methods

#### Confirmatory Analysis

No confirmatory statistical analysis will be performed. The MTD is determined according to the traditional dose-escalation scheme for Phase I trials in oncology.

#### Descriptive Analysis

Descriptive summary tables will be presented on baseline patient characteristics as well as for all safety parameters by dose level. Absolute and relative frequencies are reported for all toxicities of the CTC list (NCI CTC-AE Version 4.0) by distinguishing the grading and the assessment of the relation to treatment. Within this study, dose limiting toxicities (DLTs) are defined as any grade IV toxicity according to CTCAE Version 4.0 possibly, probably or definitely associated to study treatment during 30 days after completion of therapy.

A description of the individual load of toxicity of each patient will be made separately using individual descriptions eventually supported by graphical methods.

#### Interim Analyses

Besides the planned analysis during the dose escalation, no further interim analyses are planned.

### Data Handling, Storage and Archiving of Date

According to the §13 of the German GCP-Regulation all important trial documents (e.g. CRF) will be archived for at least 10 years after the trial termination.

According to the §28c of the German X-Ray Regulation (RöV) and the §87 of the German Radiation Protection Regulation (StrlSchV) the informed consent forms including patients' consent for trial participation, application of irradiation and data transmission to the competent authority will be archived for at least 30 years after the trial termination.

The Study Center at the Department of Radiation Oncology Define will be responsible for archiving all relevant data.

#### Good Clinical Practice (GCP)

The procedures set out in this trial protocol, pertaining to the conduct, evaluation, and documentation of this trial, are designed to ensure that all persons involved in the trial by Good Clinical Practice (GCP) and the ethical principles described in the applicable version of the Declaration of Helsinki (2008 Version of the Declaration of Helsinki, adopted at the 59th WMA General Assembly, Seoul, October 2008).

The trial will be carried out in keeping with local legal and regulatory requirements.

#### Ethics, informed consent and safety

A positive Ethics Vote was obtained by the Local Ethics Committee of the medical Faculty at the University of Heidelberg, Germany.

Before study recruitment, a positive vote of the Bundesamt für Strahlenschutz (BfS) has been obtained.

Before being admitted to the clinical trial, the subject must consent to participate after the nature, scope, and possible consequences of the clinical trial have been explained in a form understandable to him or her. The subject must give consent in writing.

#### Treatment at tumor progression

For tumor progression, treatment alternatives will be evaluated and discussed interdisciplinary considering options of surgical resection, systemic treatment such as chemotherapy, a second course of radiation therapy, or other.

## Discussion

Carbon ion radiotherapy offers distinc physical as well as biological benefits and has shown to be a promising treatment alternative for patients with HCC. Previous studies published by Japanese particle therapy centers have shown excellent results with respect to local control as well as overall survival in patients with HCC treated with carbon ion radiotherapy. In the present trial, the Phase I dose escalation concept will evaluated th optimal dose of carbonion radiotherapy for the treatment of HCC when delivered by intensity-controlled rasterscanning.

## Abbreviations

BfS: Bundesamt für Strahlenschutz; CTV: Clinical Target Volume; DLZ: Dose Limiting Toxicity; EC: Ethic's Committee; GCP: Good Clinical Practice; GTV: Gross Tumor Volume; Gy E Gray Equivalent; HCC: Hepatocellular Carcinoma; MTD: Mean tolerable dose; PTV: Planning Target Volume.

## Competing interests

The authors declare that they have no competing interests.

## Authors' contributions

SEC, JD, LE, TG and JS have developed the study concept. SEC, JD and LE wrote the study protocol and obtained ethics approval. SEC, DH, TG, JS, and JD provide patient care. TH and OJ will perform treatment planning and beam application for carbon ion radiotherapy. SEC, DH and JD will implement the protocol and oversee collection of the data. All authors contributed to and approved the final manuscript.

## Pre-publication history

The pre-publication history for this paper can be accessed here:

http://www.biomedcentral.com/1471-2407/11/67/prepub
